# Combined Targeting of Estrogen Receptor Alpha and XPO1 Prevent Akt Activation, Remodel Metabolic Pathways and Induce Autophagy to Overcome Tamoxifen Resistance

**DOI:** 10.3390/cancers11040479

**Published:** 2019-04-04

**Authors:** Eylem Kulkoyluoglu-Cotul, Brandi Patrice Smith, Kinga Wrobel, Yiru Chen Zhao, Karen Lee Ann Chen, Kadriye Hieronymi, Ozan Berk Imir, Kevin Duong, Caitlin O’Callaghan, Aditi Mehta, Sunati Sahoo, Barbara Haley, Hua Chang, Yosef Landesman, Zeynep Madak-Erdogan

**Affiliations:** 1 Department of Food Science and Human Nutrition, University of Illinois, Urbana-Champaign, Urbana, IL 61801, USA; kulkoyl2@illinois.edu (E.K.-C.); brandis2@illinois.edu (B.P.S.); kinga.wrobel@hotmail.com (K.W.); yiruster@gmail.com (Y.C.Z.); kadriye@hieronymi.de (K.H.); 2 Illinois Informatics Institute, University of Illinois, Urbana-Champaign, Champaign, IL 61820, USA; 3 Division of Nutritional Sciences, University of Illinois, Urbana-Champaign, Urbana, IL 61801, USA; kchen9282@gmail.com; 4 School of Molecular and Cellular Biology, University of Illinois, Urbana-Champaign, Urbana, IL 61801, USA; berk.imir@gmail.com (O.B.I.); kduong6@illinois.edu (K.D.); 5 Cancer Center at Illinois, University of Illinois, Urbana-Champaign, Urbana, IL 61801, USA; caitlinocallaghan22@gmail.com (C.O.); aditirmehta00@gmail.com (A.M.); 6 Beckman Institute for Advanced Science and Technology, University of Illinois at Urbana-Champaign, Urbana, IL 61801, USA; 7 UTSW Medical Center, Dallas, TX 75390, USA; sunati.sahoo@utsouthwestern.edu (S.S.); barbara.haley@utsouthwestern.edu (B.H.); 8 Karyopharm Therapeutics, Newton, MA 02459, USA; hchang@karyopharm.com (H.C.); ylandesman@karyopharm.com (Y.L.); 9 National Center for Supercomputing Applications, University of Illinois, Urbana-Champaign, Urbana, IL 61801, USA; 10 Carl R. Woese Institute for Genomic Biology, University of Illinois, Urbana-Champaign, Urbana, IL 61801, USA

**Keywords:** breast cancer, endocrine resistance, nuclear transport pathways, XPO1, ERα, metabolic rewiring

## Abstract

A majority of breast cancer specific deaths in women with ERα (+) tumors occur due to metastases that are resistant to endocrine therapy. There is a critical need for novel therapeutic approaches to resensitize recurrent ERα (+) tumors to endocrine therapies. The objective of this study was to elucidate mechanisms of improved effectiveness of combined targeting of ERα and the nuclear transport protein XPO1 in overcoming endocrine resistance. Selinexor (SEL), an XPO1 antagonist, has been evaluated in multiple late stage clinical trials in patients with relapsed and/or refractory hematological and solid tumor malignancies. Our transcriptomics analysis showed that 4-Hydroxytamoxifen (4-OHT), SEL alone or their combination induced differential Akt signaling- and metabolism-associated gene expression profiles. Western blot analysis in endocrine resistant cell lines and xenograft models validated differential Akt phosphorylation. Using the Seahorse metabolic profiler, we showed that ERα-XPO1 targeting changed the metabolic phenotype of TAM-resistant breast cancer cells from an energetic to a quiescent profile. This finding demonstrated that combined targeting of XPO1 and ERα rewired the metabolic pathways and shut down both glycolytic and mitochondrial pathways that would eventually lead to autophagy. Remodeling metabolic pathways to regenerate new vulnerabilities in endocrine resistant breast tumors is novel, and given the need for better strategies to improve therapy response in relapsed ERα (+) tumors, our findings show great promise for uncovering the role that ERα-XPO1 crosstalk plays in reducing cancer recurrences.

## 1. Introduction

The nuclear hormone receptor estrogen receptor alpha (ERα) is present in approximately 70% of both early and late stage human breast cancers [1,2]. ERα is targeted by endocrine therapies that are well tolerated and provide long-term disease-free survival if patients have localized disease [1]. Unfortunately, 30% of patients with ER positive (ER (+)) disease experience recurrence and metastasis, and there is a consistent long-term risk of death due to recurrent breast cancer with an even greater risk after 7 years [3,4,5,6,7]. Moreover, the 5-year relative survival of patients with ER (+) metastatic disease is 24%- almost none are cured and each year more women with recurrent ER (+) metastatic tumors die compared to women with ER (−) tumors [2,8]. 

Endocrine therapy is regarded as an effective treatment option for ER (+) metastatic cancer. Unfortunately, endocrine-resistance develops during the course of initial and subsequent endocrine treatment in almost all patients and occurs through various mechanisms including the mutational alterations in the ESR1 gene sequence, dysregulation in signaling pathways (i.e., Her2 signaling) and changes in drug uptake and metabolism [9]. Endocrine-resistant patients require increasingly toxic therapies. These are not optimal in terms of pharmacological properties due to poor tolerance and side effects that decrease the quality of the patient’s life. Thus, endocrine resistance remains a significant clinical problem. A novel therapeutic approach to resensitize ER (+) metastatic tumors to endocrine therapies together with methods to select patients likely to benefit from this approach is needed. Without new strategies, many patients with ER (+) tumors will continue to face diminished prospects for long-term survival being prescribed regimens that decrease their quality of life without receiving clinical benefit.

Tamoxifen (TAM) remains the “first-in-line” endocrine therapy and is regarded as one of the most effective treatments for patients diagnosed with ER (+) breast cancer. While several recent trials showed that aromatase inhibitors (AIs) in combination with ovarian suppression were effective in premenopausal breast cancer treatment, the American Society for Clinical Oncology (ASCO) continues to recommend TAM for premenopausal women [5]. This may be attributed to the observation that AIs may also have significant adverse side effects in some postmenopausal women, such as increased joint pain, bone fractures, and risk of cardiovascular disease [1,5,10]. Therefore, TAM remains as an important endocrine therapeutic agent in both pre- and post-menopausal women and is expected to continue to be widely used for some time. 

In our previously published study, which sought to understand the mechanism of TAM resistance in a preclinical setting, we identified a group of nuclear transport proteins, including exportin 1 (XPO1), which were upregulated in TAM-resistant cell lines and tumors [11]. Importantly, XPO1 upregulation caused nuclear export of ERK5, which is necessary in the nucleus for proper transcriptional response of ERα to TAM. Moreover, when ERK5 translocates to the cytoplasm, it can partner with other cytoplasmic proteins, including actin reorganization proteins that contribute to cancer cell motility. However, it is not known whether XPO1 activates other mechanisms that may be targeted by therapies in current use, specifically for endocrine therapy resistant and ER (+) breast cancers. 

Based on previous studies, [11,12,13], we hypothesized that ERα and XPO1 work together to drive TAM-resistance, and that combined targeting would more effectively sustain tumor regression than targeting either protein alone. Specifically, we showed that XPO1 mRNA levels were higher in Luminal B subtype of tumors which are more refractory to endocrine treatments, and that high XPO1 expression values were associated with a poor outcome in all women who were treated with TAM [11]. Therefore, XPO1 could be an excellent therapeutic target in breast cancer as XPO1 is already targeted in other therapy-resistant cancers with the highly specific small molecule inhibitor SEL, which is an orally active and well-tolerated drug [14,15]. Our rationale is that targeting XPO1 together with ERα is clinically relevant and combining TAM with SEL potentially offers higher efficacy, specificity, and lower toxicity for the treatment of endocrine resistant, recurrent ER (+) breast cancer. 

To evaluate the effects of combining TAM (4-OHT) with SEL, in the present study we used transcriptome analysis and found that the combination differentially modulated Akt signaling-associated gene expression as well as glycolytic and mitochondrial gene expression programs. Because of differential changes in Akt phosphorylation with 4-OHT and/or SEL, we hypothesized that metabolic pathways associated with Akt activity and consequently the metabolic profile of breast cancer cells would change in the presence of 4-OHT and SEL. We demonstrated that combined targeting of XPO1 and ERα in TAM-resistant cell lines shuts down metabolic pathways that would eventually lead to autophagy. Our studies provide a novel mechanism of action of combined targeting of ERα and XPO1 to overcome TAM resistance in recurrent ERα (+) breast cancer. 

## 2. Materials and Methods

### 2.1. Cell Culture and Ligand Treatments

All cell lines were obtained from American Type Culture Collection (Waltham, MA, USA). MCF-7 cells were cultured in Improved Minimal Essential Medium (MEM) with NEAA salts (Sigma, St Louis, MO, USA), supplemented with 10% fetal bovine serum (FBS) (HyClone, Logan, UT, USA), 100 µg/mL penicillin/streptomycin (Invitrogen, Carlsbad, CA, USA) and 1500 mg/L sodium bicarbonate (Gibco, MA, USA). *Acquired resistance* was studied from resistance progression cell lines previously derived and characterized by long-term exposure of parental MCF-7 cells to 4-OH-Tam (MCF-7 Tam^R^) [11,16]. These cells retain ERα expression, do not require E2 for growth, are not growth inhibited by SERMs and 4-OH-Tam stimulates their growth [11,16]. Human breast cancer cell lines BT474, HCC-1500 and MDA-MB-134, which are 4-OH-Tam-resistant, were used as a model of *de novo resistance* [11,17,18]. BT474 were cultured in ATCC recommended Hybri-care medium with 10% inactivated FBS, sodium bicarbonate and antibiotics. MDA-MB-134 cells were cultured in Leibovitz’s medium with 20% FBS (HyClone), and 100 g/mL penicillin/streptomycin (Invitrogen). HCC-1500 cells were cultured in ATCC-formulated RPMI1640 media with 10% FBS, sodium bicarbonate and antibiotics. Before ligand treatments, cells were grown in the corresponding phenol red-free media at least for three days. 

For isobologram analysis, BT474 and MCF-7 Tam^R^ cells were seeded at a density of 2 × 10^3^ cells/well in a 96-well plate, and were grown overnight in IMEM media without phenol red. On day 1, cells were treated with different doses of 4-OHT (from 10^−9^ M to 10^−5^ M) (Sigma) and SEL (from 10^−9^ M to 10^−5^ M) (Selleckchem, Houston, TX, USA) alone and in combination. Treatment was repeated again on day 4. On day 7, WST1 assay (Roche Molecular Systems, Inc., Pleasanton, CA, USA) was used to quantify cell proliferation ratio. Absorbance was measured at 450 nm using a Cytation5 plate reader (BioTek, Winooski, VT, USA). IC_50_ and isobologram calculations were made based on literature (*) by using Microsoft^®^ Office Excel (Seattle, WA, USA) and statistical analyses were done by using Graphpad^®^ Prism8 software (GraphPad Software Inc., La Jolla, CA, USA, www.graphpad.com). All experiment conditions had six technical repeats and experiments were repeated at least for three times.

### 2.2. RNA Sequencing Analysis

RNASeq analysis was performed as previously described [19,20,21]. Briefly, BT474 cells were treated with Vehicle (Veh, 0.5% EtOH), 10^−6^ M 4-OHT (Sigma), 10^−7^ M Selinexor (SEL) (Selleckchem) or 4-OHT+SEL combination for 24 h. Concentrations of ligands are based on our previously published study [11] and clinical data [22,23,24]. Total RNA was extracted with TRIzol reagent (Life Technologies, Carlsbad, CA, USA) according to the manufacturer’s protocol and cleaned using a clean-up kit (QIAGEN, Hilden, Germany). RNA quality was assessed using bioanalyzer. Total RNA from each sample (three per treatment group) were sequenced at the UIUC sequencing center, and data was generated in Fastqc file format to compare the expressions between the four treatment groups.

*Preprocessing and Quality Control*: Fastqc files containing raw RNA sequencing data were trimmed using Trimmomatic (Version 0.36) [25]. Next, the reads were mapped to the *Homo sapiens* reference genome (GRCh37) from the Ensembl [26] database and aligned using the STAR alignment tool (Version 2.5.3) [27]. After this, the read counts were generated from SUBREAD (Version 1.5.2) [28] and featured counts were exported for statistical analysis in R. Quality control and normalization was conducted in R using edgeR (Version 3.20.9) [29]. 

*Statistical Analysis and DEGs*: Statistical analysis was conducted in R using limma (Version 3.34.9) [30,31]. Empirical Bayesian statistics were conducted on the fitted model of the contrast matrix. Differentially expressed genes were then determined by fold-change and *p*-value with Benjamini and Hochberg [32] multiple test correction for each gene, for each treatment relative to the vehicle control. We considered genes with fold-change >1.5 and *p*-value < 0.05 as statistically significant, differentially expressed. Cluster3 software was used for clustering the differentially expressed genes. Data was visualized using Treeview Java. PCA analysis was performed using StrandNGS (Version 3.1.1). GSEA [33,34] analysis was used to identify GO terms associated with different treatments. 

### 2.3. In Vivo Xenograft Study, Immunohistochemistry Staining (IHC) and Data Analysis

All experiments involving animals were conducted with protocols approved by the University of Illinois at Urbana-Champaign and by the National Institutes of Health standards for use and care of animals (IACUC Protocol 14193). Tumor xenograft studies were performed using the BT474 cell line in immunocompromised female mice as previously described [11,35,36]. Briefly, 6-week-old BALB/c athymic, ovariectomized, nude female mice from Taconic Biosciences (Germantown, NY, USA) were used. After one week of acclimatization, 0.72 mg, 60-day release E2 pellets from Innovative Research of America (Sarasota, FL, USA) were implanted subcutaneously to maintain a uniform level of estrogen. The next day 2.5 × 10^7^ BT474 cells resuspended in 50% PBS and 50% Matrigel were injected subcutaneously into both right and left flank of each mouse. Once the tumor size reached 200 mm^3^, five animals were randomized to each treatment group. Half of the mice were implanted with vehicle pellets and the other half were implanted with 25 mg, 60-day release TAM pellets from Innovative Research of America. Each group was randomized to Vehicle or SEL injection (20 mg/kg). Concentration was selected based on clinically relevant dose [22,23,24]. Biweekly injections were performed (Monday and Friday) for 4 weeks. Each mouse was housed individually. Animals were monitored daily by the veterinarians for any signs of starvation, dehydration, stress, and pain. Total weight, food intake, and tumor size was monitored using a digital caliper biweekly. Tumors were removed from euthanized mice at the end of the experiment or at the time when tumor size reached 1000 mm^3^ and were fixed in 10% neutral-buffered formalin, processed, and embedded in paraffin in 2 M sucrose before being frozen in cutting medium. Tissues were cut in 5 microns by using a microtome (RM1255, Leica, Wetzlar, Germany). For both pAkt S473 and pAkt T308 immunostainings, tissues were deparaffinized and hydrated through graded alcohols to water. Antigen retrieval was performed by using citrate buffer, pH 6.0 in a streamer for 1 h. Samples were blocked in hydrogen peroxide for 10 min. To remove non-specific protein staining, samples were blocked with Background buster (Innovex Biosciences, Richmond, CA, USA) for 10 min and rinsed with TBS-Tween solution, pH 7.6. Then, samples were incubated with pAkt S473 primary antibody (#4060, Cell Signaling, Danvers, MA, USA) overnight at 4 °C and with pAkt T308 primary antibody (#13038, Cell Signaling) for 1 h at room temperature at 1:100 dilution. After rinsing with TBS-Tween solution, pH 7.6., samples were stained with secondary anti-rabbit and anti-mouse HRP-Polymer (Biocare Medical, Concord, CA, USA) for 30 min. Finally, samples were incubated with DAB (Innovex, Richmond, CA, USA) for 5 min and counterstained with hematoxylin, dehydrated and mounted on slides. Results are from at least four tumors per treatment. Visualization of the samples were performed with Nanozoomer Slide Scanner (Hamamatsu, Japan) at 80× magnification and positive staining quantification was performed by NDP.view software (Ver. 2.7.39, Hamamatsu Photonics, Ichinocho, Japan). Five fields per tumor were imaged and quantified. Representative images are presented. 

For the verification of XPO-1 protein levels in patient tumor samples, BRC1021 tissue microarray from Pantomics, Inc. (Richmond, CA, USA) containing 95 cases with known ER, PR, AR, Her2, p53, EGFR, and Ki67 IHC results was used. Subtype of tumors were based on following criteria: Lum A: ER (+), Her2 (−), Ki67 < 15; Lum B: ER (+), Her2 (+) or ER (+), Her2 (−), Ki67 > 15; Her2 (+): ER (−), Her2 (+); TNBC: ER (−), PR (−), Her2 (−) [37,38]. XPO1 antibody was from Bethyl laboratories (A300-469A). BRC1021 TMA included 43 Her2, 3 LumA, 31 Lum B, 17 TNBC tumor samples. UTSW cohort included 50 Luminal A, 45 Luminal B, 48 TNBC and 35 Her2 positive/amplified breast tumors validated by a pathologist. The samples were collected under the STU 102010-051 IRB protocol number, were collected at UTSW, and affiliated hospitals. For each core, a score for XPO1 was assigned based on the signal intensity (0 = none, 1 = low, 2 = moderate, and 3 = high). 

### 2.4. Western Blot Analysis in Cell Lines

Cells were seeded on 30 mm cell culture plates at 1 × 10^5^ cells/well concentration in corresponding treatment media without phenol red which contains 5% FBS Cells were treated with 4-OHT (10^−6^ M) (Sigma) and SEL (10^−7^ M) (Selleckchem)-containing treatment media overnight. Next day, cells were collected in Lysis buffer (0.5 M EDTA, 1 M TrisHCl pH 8.1, 10% SDS, 10% Empigen, ddH_2_O) with 1× Complete Protease Inhibitor (Roche) and 1× Phosphatase Inhibitor (Thermo Scientific, Waltham, MA, USA). Samples were further processed with sonication and protein concentrations were determined by BCA assay (Thermo Scientific). Then, samples were boiled in SDS-containing loading buffer and were run in 10% precast gels (BioRad) and transferred to nitrocellulose membrane. The membrane was blocked in Blocking Buffer (Odyssey^®^, Li-Cor, Lincoln, NE, USA) and target proteins were probed with XPO1 (sc-74454, Santa Cruz Biotechnology, Dallas, TX, USA), p-Akt S473 (#4060, Cell Signaling), pAkt T308 (#13038, Cell Signaling), Akt (#9272, Cell Signaling), PARP (#9542, Cell Signaling), ATG9A (STJ98598, St. John’s Laboratory, London, UK), AMBRA-1 (STJ98593, St. John’s Laboratory) and Beclin-1 (STJ 98594, St. John’s Laboratory), antibodies in 1:1000 dilution and β-actin (Sigma SAB1305546) antibody in 1:10000 dilution. The secondary antibodies were obtained from Odyssey were used at 1:10000 dilution. The membranes were visualized by using Licor Odyssey CLx infrared imaging device and software. All results were repeated at least three times and the results were normalized according to signal, which was received from β-actin loading control. Representative blots are presented. 

### 2.5. Seahorse Metabolic Profiling Assays

MCF-7, MCF-7 Tam^R^ and BT474 cells were seeded at a density of 5 × 10^4^ and MDA-MB-134 and HCC 1500 cells were seeded at a density of 1 × 10^5^ in corresponding treatment media without phenol red in each well of the XFp Cell Culture miniplates, respectively (Seahorse Bioscience Inc., Billerica, MA, USA). The FCCP concentration was 0.5 µM for MCF-7, BT474, MDA-MB-134 and HCC 1500 cells. Next day, cells were treated with 4-OHT (10^−6^ M) (Sigma) and/or SEL (10^−7^ M) (Selleckchem) -containing treatment media overnight and the cartridges were hydrated with the calibration solution and kept in a non-CO_2_ incubator at 37 °C overnight. In parallel, a duplicate of each plate was used for cell counting to monitor cell number changes after 24 h of treatments and Seahorse data was normalized to total cell number. On the assay day, cells were washed with XF Base Media without phenol red (Seahorse Bioscience Inc.) supplemented with 10 mM L-glucose, 2 mM L-glutamine (Gibco) and 1 mM sodium pyruvate (Gibco, Waltham, MA, USA). The ECAR (mpH/min) and OCR (pmol/min) values were obtained by using Seahorse XF_p_ Cell Energy Phenotype Test Kit (Seahorse Bioscience Inc.), Seahorse XF_p_ Mito Stress Test Kit (Seahorse Bioscience Inc.), Seahorse XF_p_ Glycolytic Stress Kit (Seahorse Bioscience Inc.), and Seahorse XF_p_ Mito Fuel Flex Test Kit (Seahorse Bioscience Inc.), which were run with Seahorse XFp Analyzer (Seahorse Bioscience Inc.). Experiments were performed in triplicate and repeated at least three times. 

### 2.6. Caspase Colorimetric Protease Assay

MCF-7 and BT474 cells were seeded at a density of 2 × 10^3^ cells to a 96-well plate and in the next day, they were treated with 4-OHT (10^−6^ M) (Sigma) and SEL (10^−7^ M) (Selleckchem) alone and in combination for 24 h. The colorimetric caspase activities of Caspase 2, Caspase 3, Caspase 6, Caspase 8 and Caspase 9 were determined according to the manufacturer recommendations by using ApoTarget Caspase Colorimetric Protease Assay Sampler Kit (Invitrogen, Carlsbad, CA, USA). Changes in colorimetric density were detected by Cytation™ 5 Cell Imaging Multi-Mode Plate Reader (Biotek Instruments, Inc., Winooski, VT, USA) at 400 nm wavelength. Experiments were performed in duplicates and repeated three times.

### 2.7. Autophagy Assay 

MCF-7 and BT474 cells were seeded at a density of 2 × 10^3^ cells to 96-well plate and in the next day, they were treated with 4-OHT (10^−6^ M) (Sigma) and SEL (10^−7^ M) (Selleckchem) alone and in combination for either 24 or 48 h. Autophagosome formation was detected through GFP-labelled LC3-II protein in live cells using the Cyto-ID Autophagy Detection Kit (Enzo Life Sciences, Farmingdale, NY, USA) according to manufacturer recommendations by Cytation™5 Cell Imaging Multi-Mode Plate Reader (Biotek Instruments Inc.) at ~480 nm excitation and ~530 nm emission. In addition, Hoechst 33342 Nuclear Stain was also recorded by means of DAPI filter set at ~340 nm excitation and ~480 nm emission. For flow cytometry analysis, BT474 cells were seeded at a density of 1 × 10^6^ cells/plate, and they were grown for 48 h in IMEM media without phenol red. Next day, cells were treated with 4-OHT (10^−6^ M) (Sigma) and SEL (10^−7^ M) (Selleckchem) alone and in combination for 24 h. Negative control plates were treated with EtOH and positive control plates were also treated with Rapamycin (5 × 10^−6^ M) (CytoID) for 24 h. Following the treatment process, cells were collected and centrifuged at 1000 rpm for 5 min in 1× Assay buffer provided in the CytoID^®^ Autophagy Detection Kit (Enzo Biosciences Inc, Ann Arbor, MI, USA). Cells were resuspended again and incubated with CYTO-ID^®^ Green Detection Reagent for 30 min at dark. After this incubation time, cells were washed with 1× Assay buffer 3 times and results were analyzed with results were analyzed by BD™ LSR II Flow cytometry analyzer (BD Biosciences Inc., San Jose, CA, USA). Following analysis, results were analyzed by FCS Express 6 Flow Cytometry Software (DeNovo Software) (https://www.denovosoftware.com/site/Flow-RUO-Overview.shtml) and all experiments were repeated with three technical replicates at least three times. Experiments were performed in duplicates and repeated three times. 

For the inhibition of autophagy experiments, BT474 cells were seeded in 96-well plates at a confluency of 2000 cells/well in corresponding treatment media. At day 2, they were treated in a media with 10^−6^ M 4-OHT and 10^−7^ M SEL alone or combined in the presence or absence of 10^−5^ Chloroquine (Sigma). This treatment was repeated at day 5. Cellular viability was measured with WST1 reagent (Roche) at 450 nm wavelength and results were quantified by using Cytation5 cell imaging Multi-Mode reader (Biotek Instruments Inc.). Statistical analyses were done by using GraphPad Prism8^©^ software.

### 2.8. Cell Cycle Analysis

Flow cytometry analysis was performed as previously described [11,16,39]. Briefly, MCF-7 and BT474 cells were seeded at a density of 1 × 10^6^ cells/plate and their media was changed two times in every two days. Following an overnight treatment with 4-OHT (10^−6^ M) (Sigma) and SEL (10^−7^ M) (Selleckchem) alone and in combination, cells were collected with 0.1% FBS containing 1× PBS, washed, resuspended at 1–2 × 10^6^ cells/mL and fixed with ice cold ethanol for 24 h. Next day, cells were washed with 1× PBS and incubated in 10 ng

RNAase for one hour at room temperature. Cells were stained with 0.25% Propidium Iodide (#10008351, Cayman Chemicals, Ann Arbor, MI, USA) for one hour and results were analyzed by BD™ LSR II Flow cytometry analyzer (BD Biosciences Inc., San Jose, CA, USA). Following analysis, results were analyzed by FCS Express 6 Flow Cytometry Software (DeNovo Software, https://www.denovosoftware.com/site/Flow-RUO-Overview.shtml) and all experiments were repeated three times.

### 2.9. Statistical Analyses 

Data from all studies were analyzed using a one-way analysis of variance (ANOVA) model to compare different ligand effects, a two-way-ANOVA model to compare time-dependent changes. All data was tested for normal distribution using pairwise t tests with a Bonferroni correction. All data was normally distributed unless otherwise noted. Normally distributed data was analyzed using pairwise t tests with a Bonferroni correction to identify treatments that were significantly different from each other (* *p* < 0.05, ** *p* < 0.01, *** *p* < 0.001, **** *p* < 0.0001). For every main effect that was statistically significant at α = 0.05, pairwise *t*-tests were conducted to determine which ligand treatment levels were significantly different from each other. For these *t*-tests, the Bonferroni correction was employed to control experiment wise type I error rate at α = 0.05 followed by Bonferroni post hoc test. Data that were not normally distributed were analyzed using Mann Whitney test for nonparametric data (* *p* < 0.05, ** *p* < 0.01, *** *p* <0.001, **** *p* < 0.0001). Statistical significance was calculated using GraphPad Prism for Windows. 

### 2.10. Availability of Data and Materials

Gene expression data is submitted to GEO database under the accession number GSE112883 and going to be available from the day of the acceptance of the manuscript. 

## 3. Results

### 3.1. 4-OHT+SEL Treatment Synergistically Sustains Tumor Regression in Endocrine Resistant Luminal B Subtype Breast Cancer Cells

**Figure 1:**
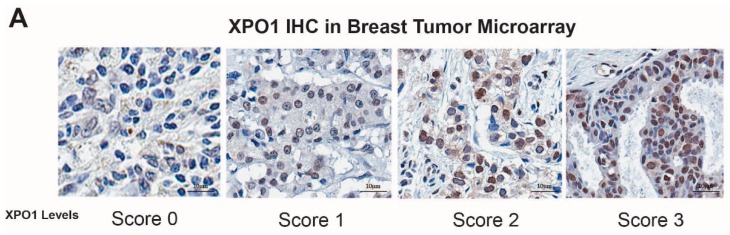

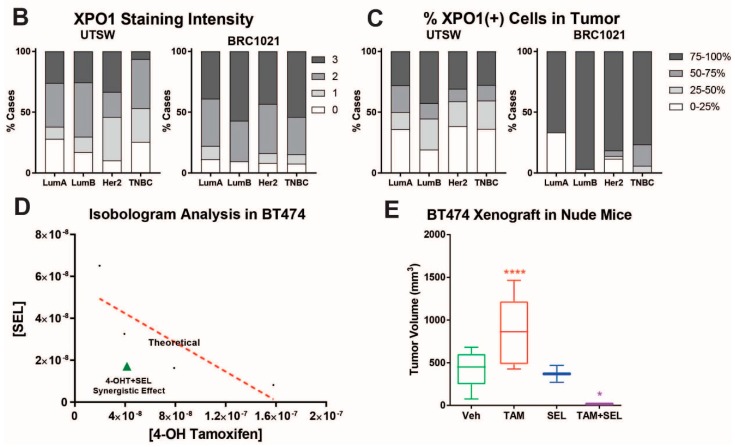
XPO1 is overexpressed in Luminal B breast cancers and targeting XPO1 modulates Akt signaling. (**A**) Verification of XPO-1 protein levels in patient tumor samples (BRC1021 from Pantomics, Inc containing 95 cases with known ER, PR, AR, Her2, p53, EGFR, and Ki67 IHC results). For each core, a score for XPO1 was assigned based on the signal intensity (0 = none, 1 = low, 2 = moderate, and 3 = high). (**B**) XPO1 staining intensity from (**A**). (**C**) Percentage of XPO1 positive cells from (**A**). (**D**) Isobologram analysis of synergy between 4-OHT and SEL in BT474 cells. (**E**) BT474 xenograft experiment showing that combined targeting of ERα and XPO1 provided sustained tumor regression. (Adapted from [11]). * *p* < 0.05, **** *p* < 0.0001

Our previous study showed that XPO1 mRNA was overexpressed in the Luminal B subtype of tumors, which are more likely to recur on endocrine treatments relative to the Luminal A subtype [11]. To validate our results, we performed IHC analysis for XPO1 protein expression in two independent breast tumor sets with samples from various breast cancer subtypes. This analysis showed that Luminal B subtype tumors have higher overall XPO1 signal intensity and percentage XPO1 positive cells in two independent cohorts of breast tumors (Figure 1A,B). We then treated endocrine resistant BT474 cells with 4-OHT, SEL or both in combination to assess whether these treatments synergized to reduce cell viability. The isobologram analysis showed that the 4-OHT and SEL combination synergized to achieve a greater decrease in cell viability than with either agent alone (Figure 1D). These results are consistent with our BT474 xenograft studies in nude mice, which showed that the tamoxifen (TAM) and SEL combination provided complete tumor regression, which was sustained several weeks after cessation of drug administration (Figure 1E) [11].

### 3.2. 4-OHT+SEL Combination Treatment Causes Gene Expression Changes Distinct from 4-OHT and SEL Treatments Alone in TAM-Resistant Cell Lines

**Figure 2:**
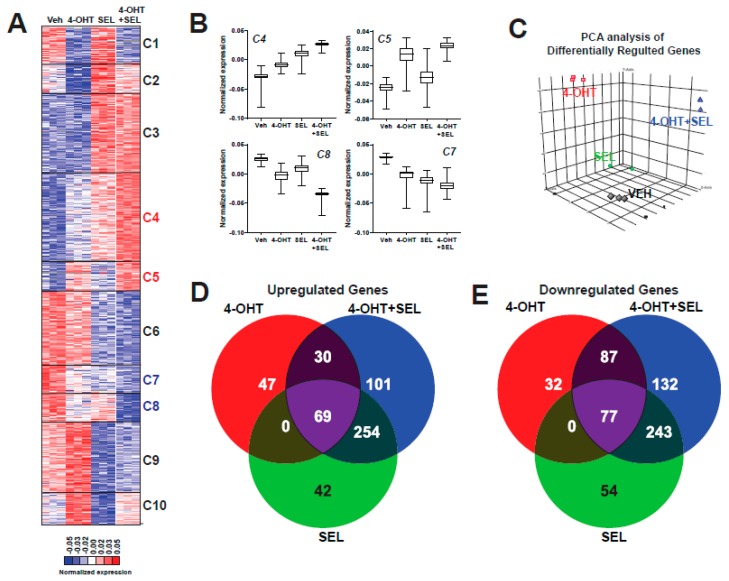
The 4-OHT+SEL combination treatment causes gene expression changes distinct from 4-OHT or SEL treatments alone in TAM resistant cell lines. (**A**) Hierarchical clustering of differentially expressed genes. (**B**) Average expression profiles of clusters that showed differential regulation by the 4-OHT+SEL combination. (**C**) PCA analysis Venn diagram analysis of Upregulated (**D**) and Downregulated (**E**) genes by different ligands.

To understand how the TAM+SEL combination provide a sustained tumor regression, we performed a global gene expression analysis in BT474 cells that were treated with Veh, 4-OHT, SEL or 4-OHT+SEL. Hierarchical clustering of the differentially-expressed genes revealed 10 clusters with different gene regulation patterns. Genes in Clusters 1, 2, 3, 6, 9 and 10 were regulated similarly with all treatments. The 4-OHT+SEL combination and 4-OHT alone resulted in similar patterns of regulation for genes in Cluster 1. The 4-OHT+SEL combination and SEL alone caused similar gene regulation patterns for genes in Clusters 2, 3, 6, 9 and 10. On the other hand, Clusters 4 and 5 include genes that were upregulated more with the combination treatment than either treatment alone, and genes in Clusters 7 and 8 were downregulated more with the combination treatment than either treatment alone (Figure 2A,B). Principle component analysis (PCA) showed that treatment groups differed significantly (Figure 2C). Venn-diagram analysis of up- and down-regulated genes revealed that the combination treatment caused gene expression changes similar to as well as different from single agent treatments. The combination treatment increased the expression of 101 genes and decreased the expression of 132 genes that were not affected by either treatment alone (Figure 2D,E).

Next, we used Gene Set Enrichment Analysis (GSEA) analysis to identify functional gene groups that were associated with different treatments (Supplementary Figure S1). The 4-OHT treatment caused upregulation in genes for cell cycle-, endocrine therapy resistance-, and breast cancer invasiveness-related pathways. On the other hand, genes involved in apoptosis and decreased tumor invasiveness were downregulated by this treatment.

Although SEL was effective in decreasing endocrine therapy resistance, breast cancer invasiveness, and metastasis related genes, genes that would regulate apoptosis were also downregulated by this treatment. The 4-OHT+SEL combination was the most effective treatment for down regulating therapy resistance and tumor invasiveness. Specifically, the combination treatment was more efficient than either treatment alone to upregulate gene sets associated with genes downregulated in Luminal B tumors, metastasis, and in bone metastasis. However, the combination treatment was very effective in downregulating ERα target genes that were upregulated by AKT1 overexpression and genes that were upregulated in Luminal B tumors, endocrine therapy resistance and metastasis that were identified in various studies. These findings are consistent with our data showing that differential Akt signaling was modulated by the ERα and XPO1 crosstalk. Of note, 4-OHT+SEL reduced targets of FGFR1 signaling, recently shown to be associated with Palbociclib + Fulvestrant-resistant tumors [40,41]. Overall, our gene expression analysis showed that the 4-OHT+SEL treatment was very effective in downregulating genes associated with endocrine resistance and metastasis. Data on other gene sets associated with metabolic regulation and autophagy will be presented in the upcoming sections. 

### 3.3. XPO1 Inhibition Modulates Differential Akt Phosphorylation in TAM-Resistant Cells and Tumor Xenografts

**Figure 3:**
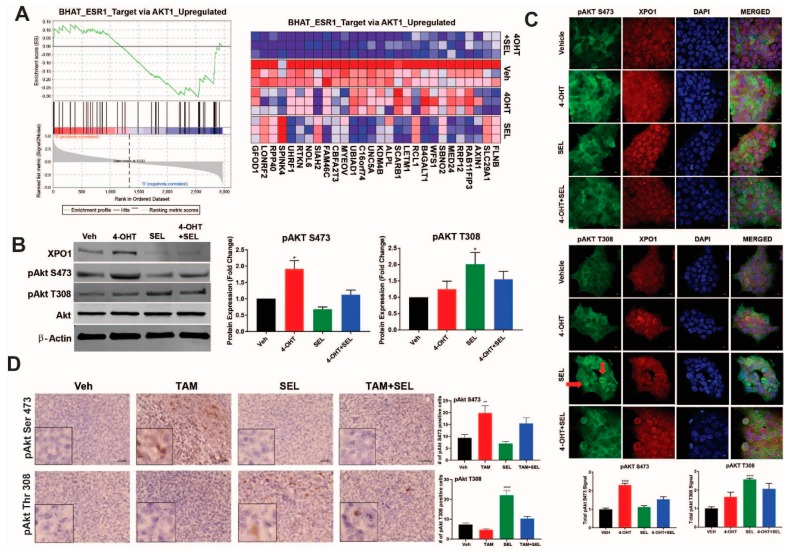
XPO1 inhibition modulates differential Akt phosphorylation in TAM-resistant cells and tumor xenografts. (**A**) GSEA analysis of RNA-Seq data depicting regulation of Akt signaling by the 4-OHT+SEL combination. ERα-XPO1 targeting changed XPO1 and pAkt protein expressions in both TAM-sensitive and TAM-resistant breast cancer cell lines. Western blot analysis (**B**) and immunofluorescence analysis (* *p* < 0.05) (**C**) of BT474 cells treated with 4-OHT (10^−6^ M) and SEL (10^−7^ M) for 24 h alone and in combination. Protein expression was quantified and is shown in bar graphs. A one-way analysis of variance (ANOVA) model was used for statistical significance of treatment effect and values were presented as mean ± SEM from three independent experiments (* *p* < 0.05, ** *p* < 0.01). scale bar = 200 pixels. (**D**) IHC staining of pAkt T308 and pAkt S473 in tumor samples obtained from BT474 xenografts received individual and combined TAM and SEL treatments (from Figure 1C). In the figure, red-brownish staining represents pAkt T308 and pAkt S473 proteins. This figure showed that individual 4-OHT and SEL treatments activated two different phosphorylation sites of Akt protein. The top panel indicated that SEL treatment activated pAkt T308 phosphorylation in BT474 tumor xenografts. The bottom panel showed that TAM treatment activated pAkt S473 phosphorylation in BT474 tumor xenografts. Quantification of positive staining was performed digitally by selecting four independent regions on the sample slides and the results were given in the corresponding graphs. Statistical significance was presented as mean ± SEM by using one-way analysis of variance (ANOVA) model (** *p* < 0.01, **** *p* < 0.0001). scale bar = 20 µm.

We next focused on the Akt signaling associated gene sets which were identified as differentially downregulated by the 4-OHT+SEL combination (Figure 3A) because they play an important role in cell survival and metabolism. We validated differential Akt phosphorylation with individual or combined drug treatments in BT474 cells (Figure 3B) and MCF-7 TAM^R cells (Supplementary Figure S2). Of note, 4-OHT treatment induced an increase in the cytoplasmic pAkt Ser473 signal, whereas SEL treatment increased the pAkt Thr305 signal in dividing cells (arrows) (Figure 3C). As we previously showed, TAM treatment stimulated tumor growth in BT474 tumor xenografts, whereas SEL treatment inhibited tumor growth [11]. However, tumor growth resumed within 3 weeks after SEL treatment was stopped. By contrast, the combination of SEL and TAM not only caused a faster and more complete regression of tumors, but the regression was also sustained [11]. To validate our pathway analysis in vivo, we utilized tumor xenograft samples from this experiment (Figure 3D). 

Our IHC analysis showed that Ser 473 phosphorylation of Akt protein increased only in tumors from TAM treated animals, whereas Thr 308 phosphorylation of Akt was more prevalent in tumors from SEL treated animals. Both of the phosphorylation events were dampened in tumors from animals that were treated with the SEL+TAM combination, suggesting that the combination treatment prevented activation of survival pathways that were otherwise prominent when tumors were treated with single agents (Figure 3D). These results suggest that ERα-XPO1 crosstalk might contribute to drug resistance by regulating differential Akt signaling, which is important for survival and metabolic control. 

### 3.4. ERα-XPO1 Targeting Changes the Metabolic Phenotype of Breast Cancer Cells from An Energetic to A Quiescent Profile

**Figure 4:**
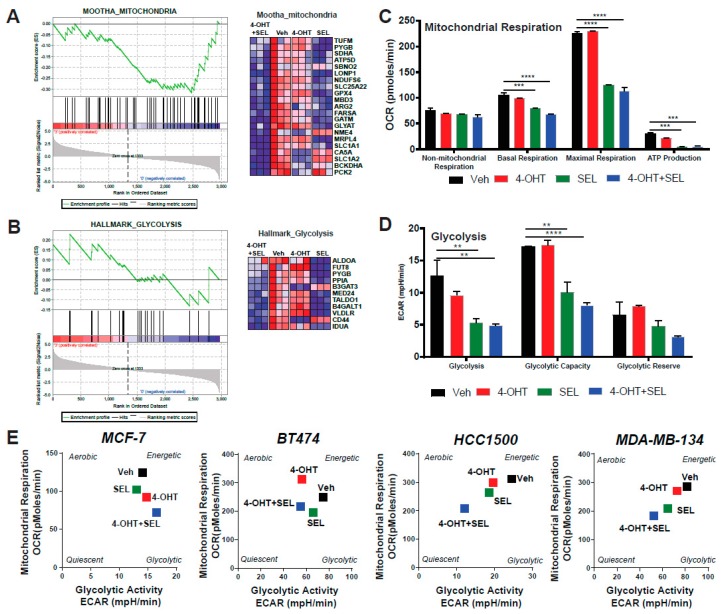
ERα-XPO1 targeting changes the metabolic phenotype of breast cancer cells from an energetic to a quiescent profile. GSEA analysis of RNA-Seq data depicting the regulation of glycolytic (**A**) and mitochondrial respiration (**B**) pathways by the 4-OHT+SEL combination. ERα-XPO1 targeting decreased the glycolytic activity of BT474 cells. Glycolytic functions were determined by the glycolysis stress test. A two-way analysis of variance (ANOVA) model was used for statistical significance of drug treatments alone or in combination and values were presented as mean ± SEM from two independent experiments and all experiments were performed in triplicates. (* *p* < 0.05, ** *p* < 0.01, *** *p* < 0.001, **** *p* < 0.0001). (**C**,**D**) ERα-XPO1 targeting decreased the mitochondrial activity of BT474 cells. Mitochondrial activity parameters were measured by the mitochondrial stress test. A two-way analysis of variance (ANOVA) model was used for statistical significance calculations and values were presented as mean ± SEM from three independent experiments (* *p* < 0.05, ** *p* < 0.01, *** *p* < 0.001, **** *p* < 0.0001). Some key parameters of mitochondrial function (Basal respiration, proton leak, maximal respiration, spare respiratory capacity, non-mitochondrial respiration, ATP production, coupling efficiency and spare respiratory capacity) were calculated and presented for each treatment conditions. (**E**) Cell phenotype assay resulted in MCF-7, BT474, MDA-MB-134 and HCC1500 cells, which were treated with 4-OHT (10−6 M) and SEL (10−7) M for 24 h alone, for 24 h alone and in combination. Changes in metabolic profile was determined by measuring oxygen consumption rate (OCR) and extracellular acidification rate (ECAR) under basal and stressed conditions with the cell phenotype assay. Values are presented as ± SEM from three independent experiments.

Our gene expression analysis also pinpointed two of the pathways associated with metabolic regulation: glycolysis and mitochondrial respiration (Figure 4A,B). Since Akt is a major regulator of cell metabolism and TAM responsiveness, we hypothesized that XPO1 modulates Akt activity to rewire metabolism and provide new survival/escape routes to breast cancer cells. We performed a mitochondrial stress test to monitor different parameters of mitochondrial respiration, including basal respiration, proton leak, maximal respiration, spare respiratory capacity, non-mitochondrial respiration, ATP production, and coupling efficiency. The 4-OHT+SEL combination did as well as or slightly better than the individual treatments in reducing different mitochondrial respiration parameters (Figure 4C). We also validated gene expression data related to metabolism using a glycolytic stress test, which monitors glycolysis, glycolytic capacity and glycolytic reserve. The 4-OHT+SEL combination was as good as or better than individual treatments in reducing all three parameters (Figure 4D). Next, we tested the individual and combinational effects of 4-OHT and SEL on the metabolic cell phenotypes of TAM-sensitive and TAM-resistant cell lines. Our results showed that although treatment with 4-OHT alone or SEL alone made all the cell lines less energetic, the cells became even more quiescent with the 4-OHT+SEL treatment (Figure 4E, Supplementary Figure S2). Of note, combining SEL with 4-OHT was as effective as combining a PI3K inhibitor (MK2206) with 4-OHT in reducing 4-OHT induced cell viability and increase in mitochondrial respiration (Supplementary Figure S3).

### 3.5. ERα-XPO1 Targeting Induces Autophagic Cell Death

**Figure 5:**
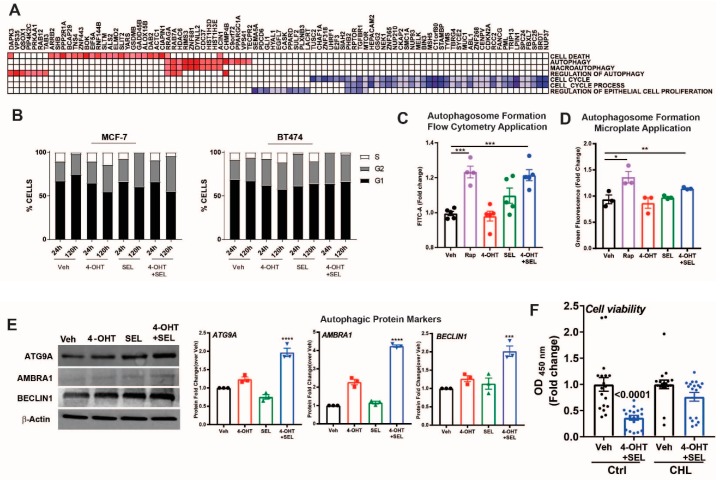
ERα-XPO1 targeting induces autophagic cell death. (**A**) GSEA analysis of cell proliferation and death pathways regulated by the 4-OHT+SEL combination treatment. (**B**) Cell cycle analysis after Veh, 4-OHT, SEL or 4-OHT+SEL treatments in MCF-7 and BT474 cells. LC3-II specific protein expression increased in TAM-resistant BT474 cells in a 24-h period. Comparison of the autophagic markers in BT474 cells treated with 4-OHT (10^−^^6^ M) and SEL (10^−^^7^ M) alone and in combination for 24 h. Autophagosome formation was detected with a colorimetric method (**C**) which measures the level of green fluorescent-labeled LC3-II specific signal or by FACS analysis of the cells after staining with antibodies specific for LC3-II (**D**). Rapamycin (50 nM) was used as a positive control for autophagy detection. A one-way analysis of variance (ANOVA) model was used for statistical significance of treatments. The experiment was performed in triplicates and values were presented as mean ± SEM from two independent experiments (*** *p* < 0.001). (**E**) The western blot analysis showed that the level of autophagic protein markers are higher in BT474 cells treated with both 4-OHT (10^−6^ M) and SEL (10^−7^ M) compared to those treated with individual 4-OHT+SEL treatment. BT474 cells were seeded at a density of 1 × 10^5^ cells/well and treated with 4-OHT (10^−6^ M) and SEL (10^−7^ M) alone and in combination. Protein expressions of autophagic proteins (ATG9A, AMBRA, ULK1 and Beclin-1) were detected with target specific antibodies at 1:1000 dilution. Protein signal was quantitated using Licor Odyssey. A one-way analysis of variance (ANOVA) model was used for statistical significance of treatment and values were presented as mean ± SEM from three independent experimental repeats. (**F**) Autophagy inhibitor chloroquine inhibited 4OHT+SEL induced decrease in cell viability. One-way analysis of variance (ANOVA) model was used for statistical significance of treatments. (* *p* < 0.05, ** *p* < 0.01, *** *p* < 0.001).

Our gene expression analyses indicated that gene sets associated with cell cycle and regulation of epithelial cell proliferation were downregulated, whereas gene sets associated with autophagy and cell death were upregulated (Figure 5A). To validate these results, we performed cell cycle analysis in MCF-7 cells and BT474 cells treated with Veh, 4-OHT, SEL or the 4-OHT+SEL combination. The combination treatment was very effective, particularly in BT474 cells, in reducing ratio of S-phase cells (Figure 5B). We also validated autophagic vacuole formation (Figure 5C,D) and an increase in autophagy related proteins in this cell line (Figure 5E). In addition, we examined different apoptotic caspase activities, PARP cleavage and changes in apoptotic cells in both cell lines. We did not observe any indicator of apoptosis in either of the cell lines at up to 120 h of treatment (Supplementary Figure S3). Finally, treatment of BT474 cells with the autophagy inhibitor chloroquine blocked the 4-OHT+SEL mediated reduction in cell viability (Figure 5F). Together, these results reveal that co-inhibition of ERα-XPO1 leads to autophagic cell death. 

## 4. Discussion 

This study is the first of its kind to report the molecular basis of the effectiveness of the novel combination therapy of 4-OHT and SEL in providing sustained tumor regression for ER (+), Tam-resistant tumors. We showed that individual 4-OHT and SEL treatments increased differential Akt signaling in endocrine-resistant cells, but that the combination prevented this activation. We also reported transcriptional and functional consequences of combined ERα and XPO1 targeting. The combination therapy was the most efficient treatment at reversing the expression of endocrine resistance and metastasis-related gene expression programs. In addition, glycolytic and mitochondrial pathways were targeted by this combination. As a result, the 4-OHT+SEL combination effectively blocked glycolysis and mitochondrial respiration and caused cell death by activating autophagy. Our study is the first documented attempt to define the causal role of metabolic programming in therapy resistant ER (+) tumors and to explore the feasibility of the combined targeting of these pathways to improve the response to endocrine agents and decrease the risk of recurrence. 

Targeting XPO1 together with ERα is clinically relevant for several reason. First, in our published analysis from publicly available tumor datasets [11] and data from patient tumor samples (Figure 1A) mRNA and protein levels of XPO1 were higher in Luminal B subtype tumors, which are more likely to recur on endocrine treatments relative to Luminal A subtype. Second, high XPO1 mRNA expression was associated with a poor outcome in women who were treated with TAM [11]. And last, XPO1 was already being targeted in phase 2 and 3 clinical trials (clinicaltrials.gov) by SEL- a specific, covalent, small molecule orally bioavailable inhibitor of XPO1, to treat patients with hematological and solid cancers [22,23,24,42,43]. SEL is well tolerated with manageable side effects including nausea, fatigue and anorexia that improve over time on treatment. Even in patients that remained on therapy for more than 8 months, no significant cumulative drug toxicities have been identified [15]. Since cancer cells of different tumor types have been shown to be more sensitive to XPO1 inhibition than normal cells [44,45], combining TAM with SEL potentially offers higher efficacy, specificity and lower toxicity for treatment of endocrine resistant, recurrent ER (+) breast cancer. 

**Figure 6:**
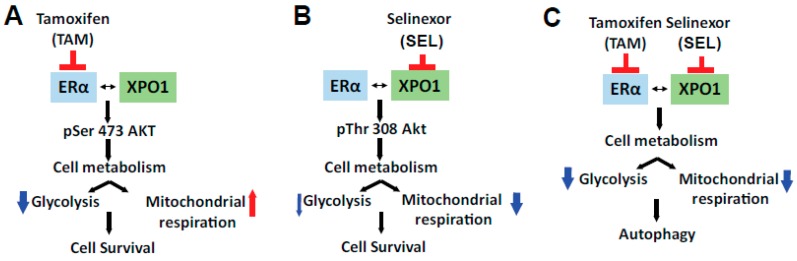
Molecular mechanism of sustained tumor regression by combined ERα-XPO1 targeting.

We propose that co-targeting ER and XPO1 is an improved therapeutic strategy because inhibiting the proteins in combination caused both a metabolic shift and induced autophagy, which together led to prolonged tumor regression. The combined targeting of ERα and XPO1 overcame TAM resistance, modulated cellular signaling to prevent rewiring of tumor cell metabolism and induced cell death by autophagy (Figure 6). We showed that single or combined targeting of ERα and XPO1 caused differential regulation of Akt phosphorylation. The Akt pathway is a master regulator of cancer cell metabolism [46], components of PI3K/AKT/mTOR pathway are mutated in nearly 25% of breast tumors and are associated with drug responses in ER (+) and ER (−) tumors [47,48,49]. Estrogens stimulate this pathway to regulate migration and invasion of cancer cells characteristic of ER (+) tumors [50,51]. In return, mTOR signaling regulates the expression level and activity of ERα and mTOR acts as a coregulator for ERα [52,53]. Inhibition of PI3K increases ER expression and activity [48,54], and PI3K inhibitor-endocrine agent combinations were tested with minor success in clinical trials to treat women with endocrine resistant disease (NCT01339442, NCT02273973). Akt signaling regulates both aerobic glycolysis and oxidative phosphorylation and impact phenotypic features of tumor cells [55]. Our pathway activation assay showed that two established Akt targets, ENOS [56,57] and PLCγ-1 [58], which are associated with angiogenesis and metastasis [59], had increased phosphorylation levels when cells were treated with SEL only but not with the 4-OHT and SEL combination. A similar pattern of regulation was observed for p53 phosphorylation at Ser15 by SEL and Ser392 by 4-OHT. Interestingly, loss of Ser392 phosphorylation by p70S6K was shown to inhibit autophagy by decreasing expression of ULK1 in the context of oxidative stress [60] whereas the same modification by p38 MAPK activated autophagy [61]. Of note, p38 MAPK phosphorylation was increased only in the 4-OHT+SEL treated cells, which might explain why we observe autophagy only in this treatment condition.

The majority of current research efforts in the therapy resistance field focuses on a delineation of the underlying mechanisms that lead to increased activity of selective signaling pathways. Undoubtedly, interrogating and targeting the end-point kinases in tumors are highly relevant and these studies lead to the development of combination therapies involving PI3K inhibitors or mTOR pathway inhibitors together with endocrine agents. However, resistance to these combination therapies also occurs, and in such cases, the cancer that develops is considerably more aggressive due to hyperactivation of compensatory mitogenic signaling pathways [62]. Moreover, these kinase inhibitors have many adverse side effects. More recently, ERα mutations that decrease sensitivity of the receptor to selective estrogen receptor modulators (SERMs) and selective estrogen receptor degraders (SERDs) were identified in about 15–40% of the metastatic, but not primary, tumors after AI treatment [63,64,65,66,67]. However, in ER (+) metastatic tumors that occur after TAM treatment, such mutations were not identified [68,69,70]. Our proposed treatment approach might prevent/delay the use of AIs and emergence of ESR1 mutations in tumors that recur after endocrine therapies, which is currently a significant clinical challenge.

Our studies described here provide a vast array of novel and important new information that will significantly advance our understanding of adaptive metabolic pathways associated with therapy resistance and cancer cell survival. The nuclear export pathways have not previously been implicated in TAM resistance and given the need for better strategies for selecting patients to receive TAM and improving therapeutic response of relapsed ERα (+) tumors, our results have great potential for uncovering the role of these pathways and their combined targeting in reducing recurrences and deaths due to metastatic ER (+) tumors. We expect our studies to establish a novel and innovative concept of combined targeting of ERα and XPO1 in TAM resistance that has not been previously explored in the field and will advance a new therapeutic strategy to the clinic. Successful translation of our findings to the clinic will change the treatment regimens for already metastasized patients while on TAM by replacing them with ones that are more effective, less toxic, and will positively impact the patient’s survival.

## 5. Conclusions

In conclusion, ERα-XPO1 crosstalk is a driver of TAM-resistance by causing mislocalization of key factors. Co-targeting XPO1 and ERα in endocrine resistant tumors resensitizes tumors to TAM and achieve complete tumor regression by inhibiting cell metabolism and inducing autophagy. However, we do not know if we can combine XPO1 inhibitors with other therapies currently used in the clinics for treatment of metastatic BCas and future preclinical studies are needed to answer these questions.

## Supplementary Materials

The following are available online at https://www.mdpi.com/2072-6694/11/4/479/s1, Figure S1: Analysis of gene functional groups associated with different ligand treatments, Figure S2: Regulation of signaling pathways by 4-OHT and SEL, Figure S3: Combined ERα-XPO1 targeting cause apoptosis neither in MCF-7 nor in BT474 cells.
