# Supplementary material for: Combined Targeting of Estrogen Receptor Alpha and XPO1 Prevent Akt Activation, Remodel Metabolic Pathways and Induce Autophagy to Overcome Tamoxifen Resistance
Source: Cancers (Basel). 2019 Apr 4;11(4):479. doi: 10.3390/cancers11040479 (PMC6520695; doi:10.3390/cancers11040479)
Supplement: Supplementary file 1 [file cancers-11-00479-s001.pdf]

cancers

Supplementary Materials: Combined Targeting of Estrogen Receptor Alpha and XPO1 Prevent Akt Activation, Remodel Metabolic Pathways and Induce Autophagy to Overcome Tamoxifen Resistance

Eylem Kulkoyluoglu-Cotul, Brandi Patrice Smith, Kinga Wrobel, Yiru Chen Zhao, Karen LeeAnn Chen, Kadriye Hieronymi, Ozan Berk Imir, Kevin Duong, Caitlin O'Callaghan, Aditi Mehta, Sunati Sahoo, Barbara Haley, Hua Chang, Yosef Landesman and Zeynep Madak-Erdogan

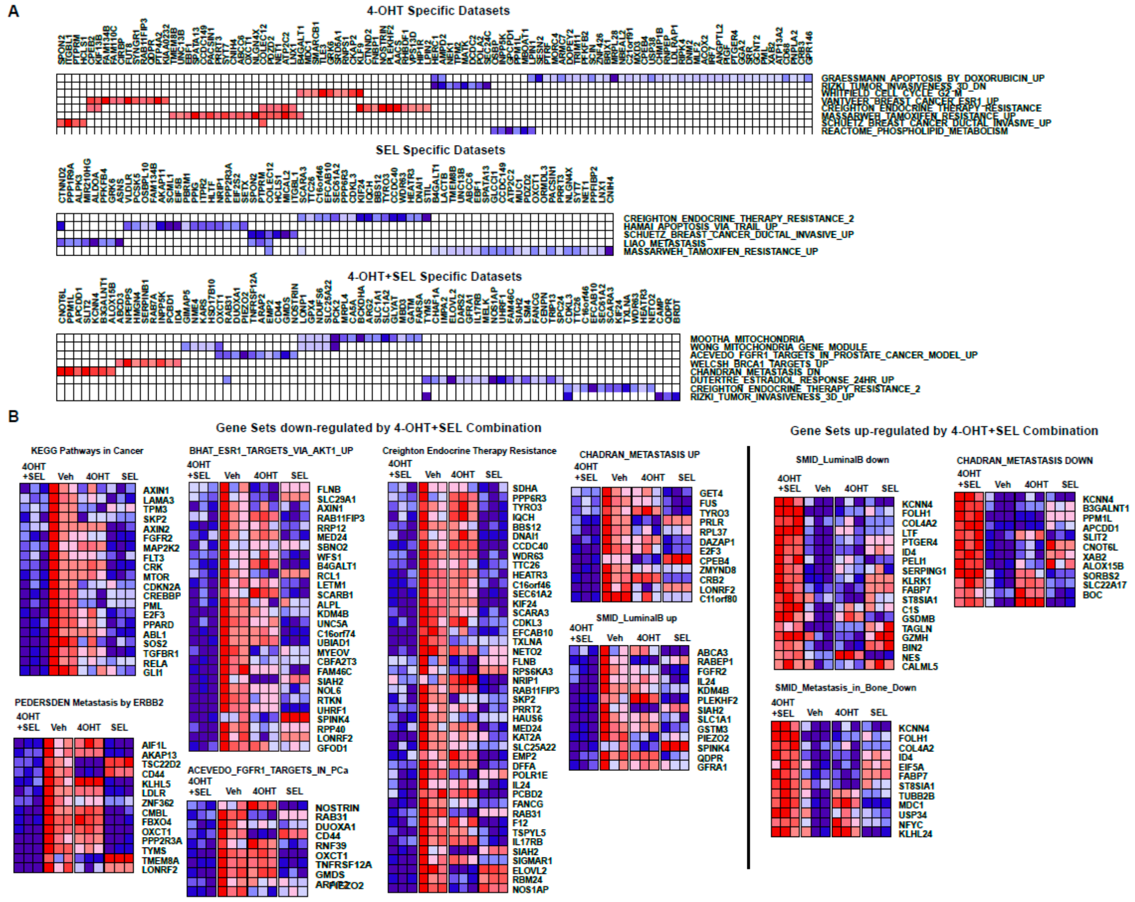

Figure S1. Analysis of gene functional groups associated with different ligand treatments. (**A**) Gene set enrichment analysis of genes that are regulated only in indicated ligand treatment group. (**B**) Gene sets that regulated greater than or equal to 4-OHT+SEL group compared to individual ligand treatments.

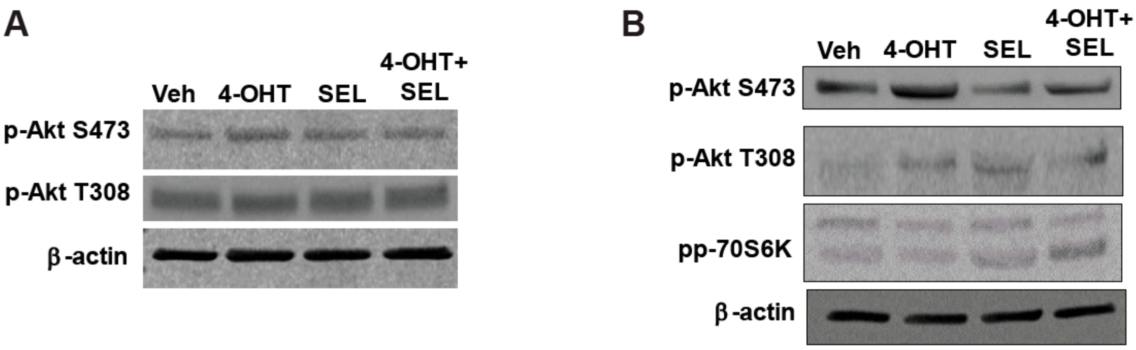

Figure S2. Regulation of signaling pathways by 4-OHT and SEL. (**A**) Western blot analysis of Akt phosphorylation in MCF-7 TAM^R cells. (**B**) Modulation of mTOR signaling by 4-OHT and SEL treatments in BT474 cells.

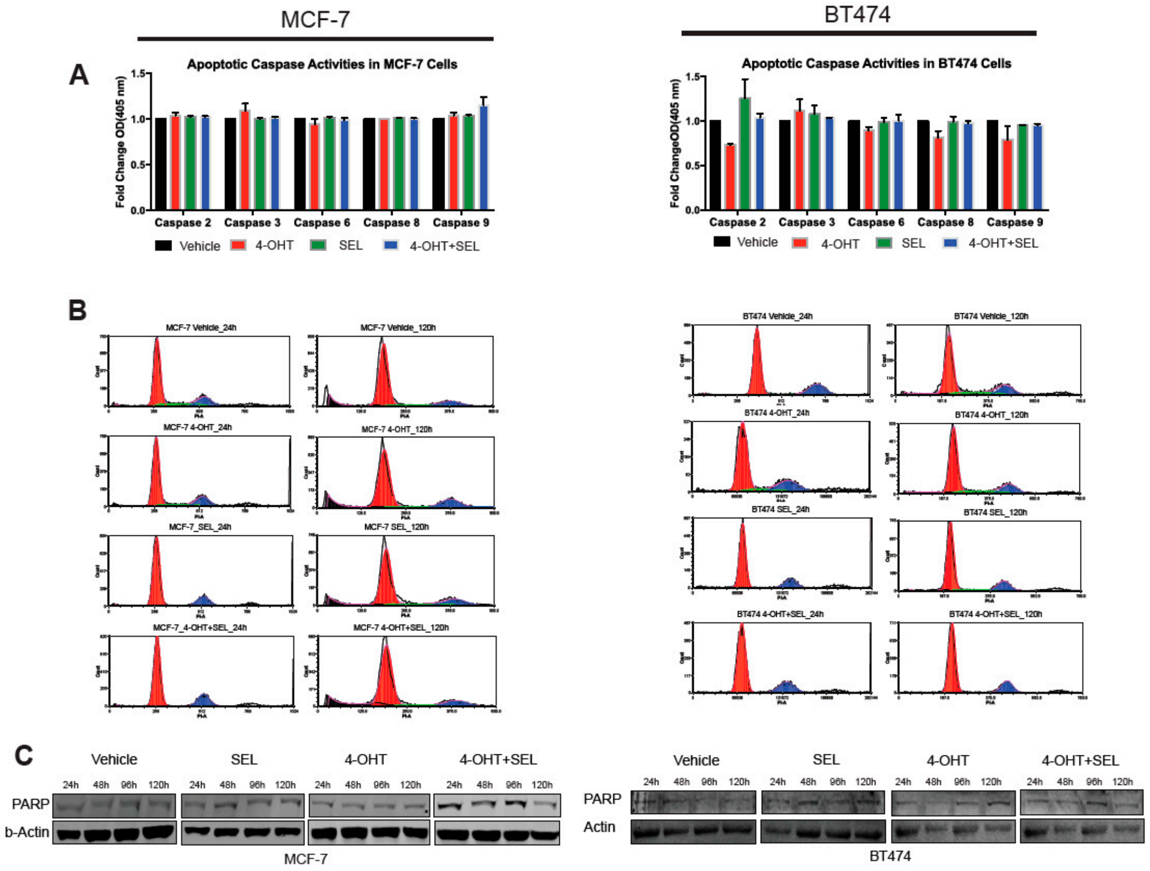

Figure S3. Combined ERα-XPO1 targeting cause apoptosis neither in MCF-7 nor in BT474 cells. (**A**) ERα-XPO1 targeting does not activate caspase protein activities. Apoptotic caspase activities of MCF-7 and BT474 cells were determined by a colorimetric method. Cells were seeded at a density of 2×10^3 cells/well and treated with 4-OHT (10^{-6} M) and SEL (10^{-7} M) alone and in combination for 24 h and different caspase activities were measured at 405 nm. Experiment was performed in triplicates and results were repeated two times. A two-way analysis of variance (ANOVA) model was used for statistical significance of ligand treatments. Values are presented as mean ± SEM from two independent experiments. (**B**) ERα-XPO1 targeting does not cause apoptotic body formation either in MCF-7 or BT474 cells. Detection of apoptosis by flow cytometer analysis. MCF-7 and BT474 cells were seeded at a density of 1×10^6 cells/well and treated with 4-OHT (10^{-6} M) and SEL (10^{-7} M) alone and in combination for 24 h and 120 h. Apoptotic body formation was indicated with cell cycle plots which were created by using FCS Express 6 (Black = Apoptotic bodies, Red = G1, Green = S, Blue = G2). (**C**)

Combined ERα-XPO1 targeting does not kill either MCF-7 or BT474 cells by apoptosis in a 96-h period. Western blot analysis of PARP protein levels in a 120-h time course. Both MCF-7 and BT474 cells were seeded at a density of 1×10^5 cells/well and treated with 4-OHT (10^{-6} M) and SEL (10^{-7} M) alone and in combination for 24, 48, 72, 96 and 120 h (Antibody dilution: 1:1000). Each treatment condition was repeated three times and the bands were quantified and analyzed by using one-way ANOVA method, but no significant different changes was seen between different treatments from 24 to 96 h.

© 2019 by the authors. Licensee MDPI, Basel, Switzerland. This article is an open access article distributed under the terms and conditions of the Creative Commons Attribution (CC BY) license (http://creativecommons.org/licenses/by/4.0/).
